# Establishment of pericentromeric heterochromatin in development

**DOI:** 10.1186/1756-8935-6-S1-O43

**Published:** 2013-03-18

**Authors:** Angele Santenard, Joanna Jachoviwcz, Maria-Elena Torres-Padilla

**Affiliations:** 1 IGBMÇ Strasbourg, France

## 

Mammalian development begins with fertilisation of an oocyte by the sperm, followed by epigenetic reprogramming of both parental genomes. Reprogramming involves de-novo establishment of chromatin domains, including resetting the characteristic features of pericentric heterochromatin, essential to progress through the first embryonic mitosis. We have determined the kinetics of incorporation of newly synthesised histones immediately after fertilisation. Our previous results showed that the histone variant H3.3 localises to paternal pericentromeric chromatin during S-phase at the time of transcription of pericentromeric repeats and that H3.3, and in particular its lysine 27, is required for the establishment of pericentromeric heterochromatin in the mouse embryo. Indeed, the mutation of H3.3K27, but not H3.1K27, results in aberrant accumulation of pericentromeric transcripts, HP1 mislocalisation, dysfunctional chromosome segregation and developmental arrest. We have now dissected both temporally and spatially the requirements for the establishment of pericentromeric heterochromatin after fertilisation. Our results suggest that the temporal ordered of events that follow fertilisation and the localisation of heterochromatin in the 3D nuclear space are tightly regulated and function in parallel to ensure heterochromatic silencing and subsequent development.

